# Weak Satiety Responsiveness Is a Reliable Trait Associated with Hedonic Risk Factors for Overeating among Women

**DOI:** 10.3390/nu7095345

**Published:** 2015-09-04

**Authors:** Michelle Dalton, Sophie Hollingworth, John Blundell, Graham Finlayson

**Affiliations:** Appetite Control & Energy Balance Research, School of Psychology, Faculty of Medicine and Health, University of Leeds, Leeds LS2 9JT, UK; E-Mails: s.l.hollingworth11@leeds.ac.uk (S.H.); j.e.blundell@leeds.ac.uk (J.B.); g.s.finlayson@leeds.ac.uk (G.F.)

**Keywords:** satiety responsiveness, satiety quotient, energy intake

## Abstract

Some individuals exhibit a weak satiety response to food and may be susceptible to overconsumption. The current study identified women showing consistently low or high satiety responses to standardised servings of food across four separate days and compared them on behavioural, psychological and physiological risk factors for overeating and future weight gain. In a crossover design, 30 female participants (age: 28.0 ± 10.6; body mass index (BMI): 23.1 ± 3.0) recorded sensations of hunger in the post-prandial period following four graded energy level breakfasts. Satiety quotients were calculated to compare individuals on satiety responsiveness across conditions. Body composition, resting metabolic rate (RMR), energy intake, food reward and craving, and eating behaviour traits were assessed under controlled laboratory conditions. A distinct low satiety phenotype (LSP) was identified with good consistency across separate study days. These individuals had a higher RMR, greater levels of disinhibition and reported feeling lower control over food cravings. Further, they consumed more energy and exhibited greater wanting for high-fat food. The inverse pattern of characteristics was observed in those exhibiting a consistently high satiety phenotype (HSP). Weak satiety responsiveness is a reliable trait identifiable using the satiety quotient. The LSP was characterised by distinct behavioural and psychological characteristics indicating a risk for overeating, compared to HSP.

## 1. Introduction

Weight gain leading to obesity is a multi-factorial problem that arises when energy intake exceeds energy expenditure over a prolonged period of time. However, the relationship between energy that is taken in and energy that is expended is not a simple one but rather involves a complex set of interactions from genetic, behavioural, physiological, social and environmental factors. Impairment in appetite control has been identified as an important contributor to overconsumption and weight gain [1,2] with one potential marker of susceptibility to overeating and obesity being a weakened satiety response to food [3]. Indeed, evidence based on clinical observations suggest some obese patients report a poor relationship between their eating patterns and their sensations of hunger and fullness, suggesting that some individuals may experience an altered or weakened recognition and response to these internal signals [4].

Research examining individual differences in satiety responsiveness has demonstrated that obese individuals who report no relationship between their eating behaviour and appetite sensations exhibited a weaker satiety response during a test meal compared to obese individuals who reported that their eating behaviour was related to their appetite sensations [5]. Interestingly, obese individuals with weak satiety responsiveness had higher scores on the Three Factor Eating Questionnaire (TFEQ) subscales of Disinhibition and Hunger compared to controls [5], which are eating behaviour traits associated with overconsumption, a higher body mass index (BMI) and when high scores on these traits are combined, opportunistic eating [6,7].

Weak satiety responsiveness is not limited to obese individuals, indeed, research led by Drapeau and colleagues have found evidence for individual variability in satiety responsiveness among both obese and normal weight individuals and have identified a “low satiety phenotype” [8,9,10]. In these studies, the authors used the satiety quotient (SQ) [11] as a psychological marker of the satiating efficiency of food (changes in sensations of hunger relative to ingested caloric load over time) to identify individuals as high or low in satiety responsiveness. The SQ represents a change in recorded appetite sensations in response to a standardised meal and is expressed per unit of intake (e.g., kcal). A low SQ in response to a fixed-energy meal has been shown to be associated with greater subsequent *ad libitum* energy intake measured under both laboratory [8,9] and free-living conditions [9]. The SQ has been used in a wide range of research and has been shown to have value in studies examining acute [12] and long term human appetite control [13] and in research examining the effects of drugs on appetite [14]. Using the SQ, research has demonstrated that the low satiety phenotype is characterised by greater levels of state anxiety and night eating symptoms, an external locus of hunger, and a blunted cortisol response to a standard test meal, suggesting the low satiety phenotype may experience some level of hypothalamic-pituitary-adrenal (HPA) axis activity dysregulation [10].

Taken together, these findings show that weak satiety responsiveness can be objectively measured using the SQ and that a weakened recognition and response to satiety signals could promote overconsumption, which over time (and in conjunction with other contributory factors) may be associated with an increase in body weight. However, previous research has only characterised individuals as low or high in satiety responsiveness using a single standardised meal consumed on one occasion. It is therefore not currently known how reliable such measures of satiety responsiveness are, or whether the low satiety phenotype is a consistent and enduring trait in response to different energy loads. In addition, it would be predicted that individuals with a weak response to internal signals would be at greater risk of overeating for hedonic reasons from external cues. However, studies have not examined whether the low satiety phenotype is associated with hedonic risk factors such as increased liking and wanting for food, and greater levels of food craving. Therefore, the first aim of the current study was to determine the consistency of the SQ to categorise women as low or high in satiety responsiveness across different energy loads. In addition, the current study sought to extend previous findings by characterising the behavioural (*ad libitum* energy intake), psychological (liking and wanting, food craving, eating behaviour traits) and physiological (body composition, resting metabolic rate) risk profile for overeating of the low satiety phenotype in a sample of normal and overweight women.

## 2. Materials and Methods

### 2.1. Participants

Thirty female participants (age: 28.0 ± 10.6; 20–54 years, BMI: 23.1 ± 3.0; 18.8–29.1 kg/m^2^) were recruited from the staff and student population at the University of Leeds, UK. Participants were selected from an initial screening process to exclude those who were taking medication known to affect appetite, currently dieting to lose or gain weight, not regular breakfast consumers, reported a history of eating disorders, or were unfamiliar with or disliked the study foods. Informed written consent was obtained prior to the study. Participants received £30 for their participation. All research procedures were reviewed and approved by the University of Leeds, School of Psychology Ethics Committee in accordance with the Helsinki declaration.

### 2.2. Design

The study followed a counterbalanced, crossover within subjects design with subjects being randomised to condition order. Participants attended the research unit on five occasions: one screening visit and four experimental visits. Each visit was scheduled at least seven days apart. For all visits, participants were asked to refrain from eating or drinking anything besides water from 10 pm the evening before to ensure a standardised fasted state In addition, participants were required to refrain from engaging in moderate to vigorous physical activity and from drinking alcohol 24-h prior to the experimental test sessions. Compliance with these instructions was assessed at the beginning of each test session by self-report. During the experimental visits, participants were permitted to leave the research unit in the period between breakfast and lunch. During the time spent outside of the research unit participants were instructed not to eat or drink anything besides water. In addition, participants were asked not to engage in moderate or vigorous physical activity at any point during the test day, but this was not objectively monitored (*i.e.*, with physical activity monitors). Ratings of subjective appetite were taken every 30 min using a validated, hand-held Electronic Appetite Ratings System (EARS II; HP® iPAQ) [15], which provided a time stamp for each entry so that compliance with this measure could be monitored. To determine whether participants were reliably low or high in satiety responsiveness changes in subjective ratings of hunger and fullness were recorded before and following consumption of four, fixed energy breakfasts. Each breakfast was individually calibrated to provide participants with graded levels of their measured resting metabolic rate (RMR), specifically, 20%, 25%, 30% and 35%.

### 2.3. Measures

#### 2.3.1. Resting Metabolic Rate

Participants’ RMR was measured during an initial screening visit following an overnight fast using an indirect calorimeter fitted with a ventilated hood (GEM; Nutren Technology Ltd., Manchester, UK). Participants were asked to remain awake but motionless in a supine position for 45 min, with RMR calculated from respiratory data averaged over the final 30 min of assessment. Oxygen uptake and maximal CO_2_ were calculated from O_2_ and CO_2_ concentrations in inspired and expired air diluted in a constant airflow of ~40 L/min (individually calibrated for each participant) and averaged over 30 s intervals. RMR was used to calibrate the fixed energy breakfasts to provide participants with graded levels of their resting energy requirements (20%, 25% 30% and 35%) in order to systematically characterise participants as high or low in satiety responsiveness.

#### 2.3.2. Anthropometrics and Body Composition

During an initial screening visit, standing height without shoes was measured to the nearest 0.5 cm using a stadiometer (Seca, Birmingham, UK). Body weight was measured using an electronic balance (Seca, Birmingham, UK) and recorded to the nearest 0.1 kg. Waist circumference (cm) was measured 1 cm above the top of the participants’ naval after expiration, two measures were taken and an average was calculated if the two were similar (within 5 mm), if they were dissimilar the entire waist circumference measure was taken again and the average of the two new measurements were recorded. Air plethysmography (Bodpod, Concord, CA, USA) was used in order to obtain an estimate of participants’ fat mass, fat free mass, and percentage body fat. Measures of body composition were taken whilst participants were wearing non-underwired swimwear and a swim cap. All measures were taken following an overnight fast.

#### 2.3.3. Subjective Appetite Sensations

Subjective appetite sensations were measured using 100 mm visual analogue scales (VAS). Measures of hunger (“how hungry do you feel now?”) and fullness (“how full do you feel right now?”) were anchored at each end with the statements “extremely” and “not at all.” Ratings of prospective consumption (“how much food could you eat right now?”) and desire to eat (“how strong is your desire to eat?”) were anchored at each end by “none at all” and “a very large amount”, and “not very strong” and “very strong,” respectively. VAS have been shown be sensitive to experimental manipulations and have good test-retest reliability [16].

#### 2.3.4. Satiety Quotient

The SQ is a measure of the satiating capacity of foods relative to energy content. The SQ has been validated in previous research [9,11]. Hunger VAS ratings were used to calculate the average SQ for the 75-min post-breakfast period (VAS taken +15 min, +45 min, +75 min post-breakfast). This was used to characterise participants as either low or high in satiety responsiveness. A higher SQ represents stronger appetite responses to the ingested food whereas a lower SQ represents a weaker response.

The following formula was used to calculate SQ:

$$\text{SQ }\left(\text{mm}/\text{kcal}\right)=\left(\frac{\text{Rating before eating episode }-\text{ mean of the }75-\text{min post}-\text{meal ratings}}{\text{Energy content of the test meal }\left(\text{kcal}\right)}\;\right)\times \;100$$

#### 2.3.5. Test Foods

##### Fixed Energy Breakfasts

The fixed energy breakfasts were comprised of muesli (muesli base, raisins, sultanas) combined with natural yoghurt, semi-skimmed milk and honey. The amount of energy provided at breakfast was individually calibrated to participants based on their measured RMR. The levels of resting energy requirements provided by the breakfasts were set to 20%, 25%, 30% and 35% RMR. The 20% RMR breakfast contained no almonds and provided a standardized base breakfast for the other conditions. The number of grams of almonds required to provide participants with 5% of their RMR was calculated and added to the standard breakfast to achieve the 25%, 30% and 35% RMR conditions. Volume was kept constant by manipulating the amount of water participants received. The average energy provided in each condition is shown in Table 1. Each participant consumed the four breakfasts in a randomised order across four test sessions. Participants were given 15-min to consume all of the food that was provided to them.

**Table 1:** Mean (standard deviation) and range energy (kcal) provided in the 20%, 25%, 30% and 35% resting metabolic rate (RMR) conditions.

Condition	Mean (SD)	Range
20%	258.8 (29.9)	209.8–328.6
25%	318.3 (34.5)	265.0–402.2
30%	392.6 (40.8)	334.0–494.2
35%	467.0 (47.6)	403.0–586.2

SD, standard error.

##### *Ad Libitum* Lunch

Energy intake was assessed using an *ad libitum* lunch test meal that consisted of tomato and herb risotto, strawberry yoghurt and garlic bread. The test meal was served 4 hours following the fixed energy breakfast. Participants were instructed to consume as much or as little as they wanted but to eat until they reached a comfortable level of fullness. Food was measured to the nearest 0.1 g and energy values were determined using food tables and manufacturer labelling.

#### 2.3.6. Food Reward: Explicit Liking and Implicit Wanting for Food

The Leeds Food Preference Questionnaire (LFPQ; [17,18]) was used to assess explicit liking and implicit wanting for an array of food images chosen to be predominantly high (>40% energy) or low (<20% energy) in fat. To measure explicit liking, the food images were presented individually, in a randomised order and participants were required to rate “*How pleasant would it be to taste some of this food now?*” on 100 mm VAS. To measure implicit wanting, participants were presented with 96 food pairs and were asked to respond as quickly and as accurately as possible according to “*Which food do you most want to eat now?*” Reaction times for all responses were recorded and used to compute mean response times for each food type after adjusting for frequency of selection and overall mean response time. For explicit liking and implicit wanting measures, the mean for low fat scores were subtracted from the mean for high fat scorers to provide an “Appeal Bias” for high fat *versus* low fat food for each outcome.

#### 2.3.7. Psychometric Questionnaires

##### Control of Eating Questionnaire

The validated Control of Eating Questionnaire (CoEQ; [19,20]) comprises 21 items that are designed to assess the severity and type of food cravings experienced over the previous 7-days. The CoEQ has four subscales; Craving Control, Craving for Savoury, Craving for Sweet and Positive Mood. The CoEQ subscales have been shown to have good internal consistency [20]. Items on the CoEQ are assessed by 100 mm VAS with items relating to each subscale being averaged to create a final score.

##### Three Factor Eating Questionnaire

The Three Factor Eating Questionnaire (TFEQ; [21]) is a 51-item scale that assesses three aspects of eating behaviour; cognitive restraint, disinhibition of eating and susceptibility to hunger. Participants were required to respond either true or false to the first 36-items, whereas the remaining items required participants to select a response from a choice of four that varied in the level of agreement with a particular statement. Responses were scored 0 or 1 and summed, with higher scores denoting higher levels of eating disturbances. The TFEQ has been shown to have good internal validity [21].

### 2.4. Procedure

Participants attended the research unit on five occasions: one initial measurement visit and four experimental test sessions. For the experimental test sessions, participants arrived at the research unit between 8:00 and 9:00 am after having fasted from 10 pm the evening before. Baseline VAS appetite ratings were completed following which participants consumed one of the four breakfasts depending on condition. Following breakfast, the second set of VAS ratings was completed and participants were able to leave the research unit but were asked to return 4 hours later for lunchtime session. During this time the EARS-II prompted completion of the VAS ratings at 30-min intervals. At the start of the lunchtime session, participants completed the LFPQ prior to the serving of the *ad libitum* test meal. Participants were given as much time as they wanted to eat lunch. VAS appetite ratings were taken at the start and after each event in the lunchtime procedure. At the end of the lunchtime session participants confirmed their next session, or if it was their final session they completed the psychometric questionnaires and were debriefed about the study aims.

### 2.5. Data Analyses

Data were analysed using SPSS version 22 (SPSS Inc., Chicago, IL, USA) for Windows and presented as means with standard deviations. Before analysis, the data were checked for normality and outliers. Pearson correlation coefficients were used to assess the relationship between psychological and behavioural variables and the satiety quotient. Independent *t* tests were used to examine the effect of satiety responsiveness on energy intake, food reward (liking and wanting fat appeal biases) and food craving. The effect of satiety responsiveness on appetite sensations was assessed using repeated measures ANOVAs. Where appropriate, Greenhouse-Geisser probability levels were used to adjust for non-sphericity. Post hoc analyses were conducted on significant interactions using *t*-tests. An α-level of 0.05 was used to determine statistical significance.

## 3. Results

### 3.1. Overall Sample Characteristics

Characteristics of age, anthropometric measures, body composition and psychometric traits for the overall sample are shown in Table 2. Of the 30 participants who completed the study, 30% were overweight, 47% were non-students, 77% were Caucasian, 10% were Asian, 7% were Latino and 6% were African American.

**Table 2:** Mean (standard deviation) and range for age, anthropometrics, body composition and psychometric trait characteristics for the overall sample.

	Mean (SD)	Range
Age (years)	28.0 (10.6)	20–54
BMI (kg/m^2^)	23.1 (2.9)	18.8–29.1
Waist (cm)	77.2 (8.1)	66.0–102.0
Body weight (kg)	62.7 (9.1)	46.3–84.7
Fat mass (kg)	19.6 (5.5)	10.8–32.3
Fat free mass (kg)	43.1 (5.2)	34.0–55.0
Body fat (%)	30.9 (5.2)	22.6–41.8
TFEQ Restraint	9.9 (5.4)	3–20
TFEQ Disinhibition	7.2 (3.2)	0–12
TFEQ Hunger	6.5 (3.4)	0–12

SD, standard error; BMI, body mass index; TFEQ, Three Factor Eating Questionnaire.

### 3.2. Validity of the Satiety Quotient as a Marker of Susceptibility

The average SQ across all RMR conditions was negatively associated with resting metabolic rate (*r* (28) = −0.383, *p* < 0.05), a greater implicit wanting fat bias (*r* (28) = −0.487, *p* < 0.01) and TFEQ disinhibition (*r* (27) = −0.417, *p* < 0.05). These preliminary associations suggest that a low SQ is associated with risk factors for overconsumption, to explore these associations further we systematically categorised subjects as high or low in satiety responsiveness.

### 3.3. Characterisation of Satiety Phenotypes

**Figure A1:**
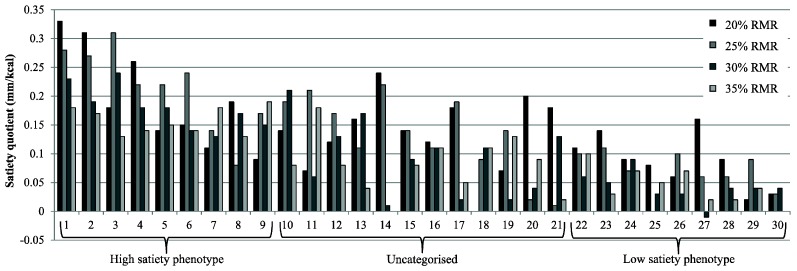
Individual variability in SQ in the 20%, 25%, 30% and 35% resting metabolic rate (RMR) conditions.

To categorise subjects as low or high in satiety responsiveness, the average SQ for the 75-min period following each breakfast was calculated. A median split for each condition was used to calculate high and low cut-off points. The low satiety phenotypes were identified as those who had a low SQ at least 3 out of 4 conditions (*n* = 9) whereas the high satiety phenotypes were identified as those who had a high SQ at least 3 out of 4 conditions (*n* = 9). Twelve participants could not be categorised as they had a low SQ in 2 conditions and a high SQ in 2 conditions, these were not included in the analysis but for interest their characteristics are shown in Table 3 (also see Figure A1).

Table 3 shows the participant characteristics for the high and low satiety phenotypes. As expected, the low satiety phenotype had a significantly lower average SQ across conditions compared to the high satiety phenotype (*t* (16) = 7.03, *p* < 0.001, *d* = 3.7). In addition, the low satiety phenotype had a higher RMR (*t* (16) = 2.33, *p* < 0.03, *d* = 1.2) and greater TFEQ Disinhibition scores (*t* (16) = 2.57, *p* < 0.02, *d* = 1.2) compared to the high satiety phenotype.

**Table 3:** Mean (standard deviation) age, appetite sensations, anthropometrics, body composition and psychometric trait characteristics for the low and high satiety phenotypes.

	Low Satiety Phenotype (*n* = 9)	High Satiety Phenotype (*n* = 9)	Uncategorised (*n* = 12)
Average SQ (mm/kcal) ^1^	6.3 (2.2) ***	18.5 (4.4) ***	11.4 (2.0)
Average baseline hunger (mm) ^1^	48.9 (15.9) ***	78.9 (10.7) ***	57.6 (14.9)
Average baseline desire to eat (mm) ^1^	48.8 (16.2) ***	78.3 (11.0) ***	56.4 (16.6)
Average baseline prospective consumption (mm) ^1^	42.7 (14.1) ***	66.3 (10.8) ***	47.3 (13.5)
Average baseline fullness (mm) ^1^	28.7 (10.8)	16.8 (12.4)	26.0 (13.5)
Age (years)	24.8 (9.1)	26.4 (10.2)	31.6 (11.6)
BMI (kg/m^2^)	24.6 (2.6)	22.7 (3.1)	22.4 (2.9)
Waist (cm)	80.9 (9.7)	74.4 (7.0)	76.4 (7.2)
Fat mass (kg)	21.5 (5.6)	19.1 (5.4)	18.5 (5.5)
Fat free mass (kg)	45.8 (6.8)	40.5 (4.2)	43.1 (3.6)
Body fat (%)	31.7 (4.4)	31.7 (5.4)	29.6 (5.7)
Resting metabolic rate (kcal)	1397.7 (185.0) *	1228.0 (116.8) *	1260.9 (104.1)
TFEQ Restraint	9.5 (5.8)	10.1 (6.4)	9.9 (4.8)
TFEQ Disinhibition	8.8 (2.2) *	5.1 (3.1) *	8.0 (3.2)
TFEQ Hunger	6.5 (3.2)	6.2 (3.7)	6.6 (3.7)

Comparisons made between low satiety phenotype and high satiety phenotype * *p* < 0.05, *** *p* < 0.001; ^1^ Average SQ, and average baseline hunger, desire to eat, prospective consumption and fullness collapsed across all resting metabolic rate (RMR) conditions; TFEQ, Three Factor Eating Questionnaire; BMI, body mass index.

### 3.4. Subjective Appetite Sensations

There was an interaction between time point and satiety responsiveness group for hunger (F (12, 144): 3.85, *p* < 0.001), desire to eat (F (12, 144): 3.08, *p* < 0.001) and prospective consumption (F (12, 144): 3.68, *p* < 0.001). Post-hoc analyses revealed that the low satiety phenotype had significantly lower baseline hunger, desire to eat, and prospective consumption scores across all conditions compared with the high satiety phenotype. There was no interaction between time point and group for ratings of fullness (F (12, 144): 1.03, *p* > 0.05).

### 3.5. Ad libitum Energy Intake

**Figure 1:**
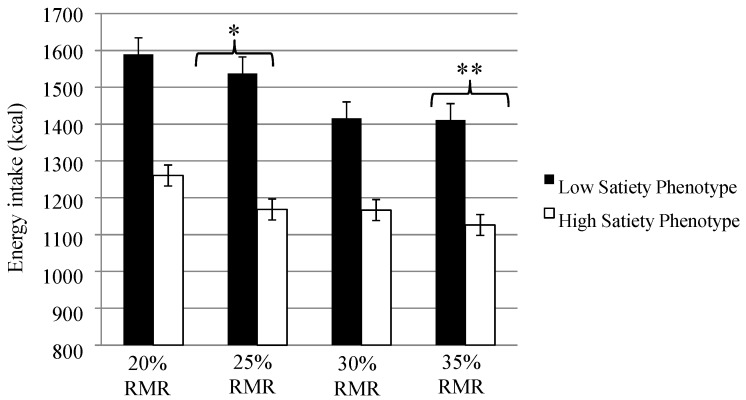
Energy intake (kcal) from the *ad libitum* lunch test meal for the low and high satiety phenotype across the 20%, 25%, 30% and 35% resting metabolic rate (RMR) conditions. * *p* < 0.05, ** *p* < 0.01.

Figure 1 shows *ad libitum* energy intake from the lunch test meal across the RMR conditions for the low and high satiety phenotype. The low satiety phenotype consumed more energy from the *ad libitum* lunch in the 25% (*t* (16) = 2.67, *p* < 0.02, *d* = 1.3) and 35% RMR (*t* (16) = 2.78, *p* < 0.01, *d* = 1.4) conditions compared to the high satiety phenotype. There were no differences in mean energy intake in the 20% RMR condition (*t* (16) = 1.97, *p* > 0.05) or the 30% RMR condition (*t* (16) = 1.36, *p* > 0.05).

### 3.6. Food Reward

#### 3.6.1. Explicit Liking Fat Appeal Bias

**Figure 2:**
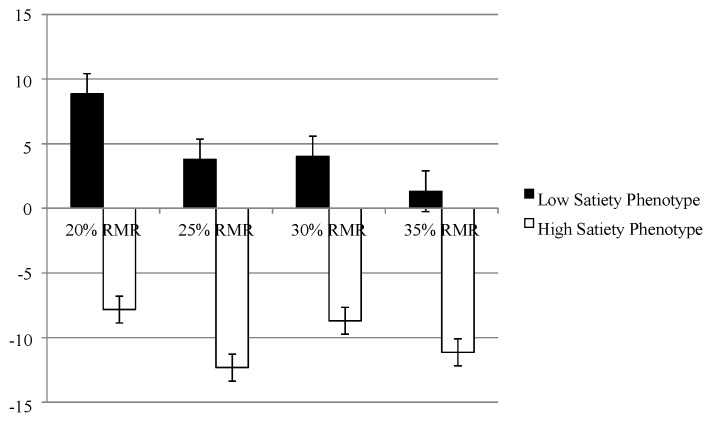
Explicit liking fat appeal biases for the low and high satiety phenotype across the 20%, 25%, 30% and 35% resting metabolic rate (RMR) conditions. A positive value indicates a bias towards high-fat foods.

Analysis of the explicit liking fat appeal bias revealed that the low satiety phenotype had a greater liking for high-fat foods when hungry compared to the high satiety phenotype who exhibited a greater bias for low-fat foods (see Figure 2). The mean differences were significant in the 20% (*t* (16): 2.32, *p* < 0.05, *d* = 1.2) and 25% (*t* (16): 2.16, *p* < 0.05, *d* = 1.1) RMR conditions but not in the 30% (*t* (16): 1.74, *p* > 0.05, *d* = 0.87) and 35% (*t* (16): 1.41, *p* > 0.05, *d* = 0.70) RMR conditions.

#### 3.6.2. Implicit Wanting Fat Appeal Bias

**Figure 3:**
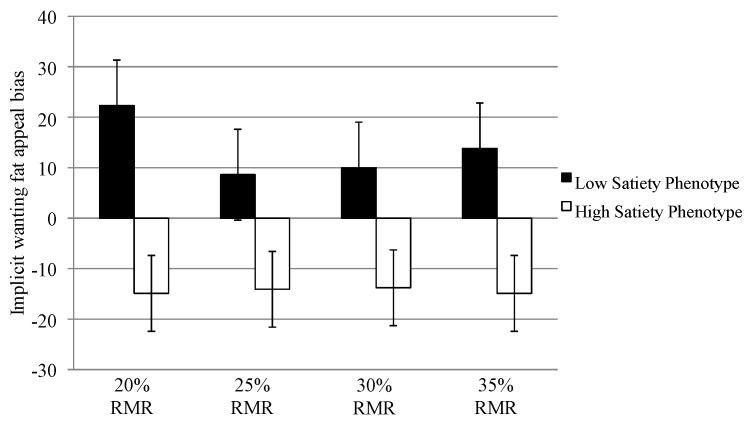
Implicit wanting fat appeal biases for the low and high satiety phenotype across the 20%, 25%, 30% and 35% resting metabolic rate (RMR) conditions. A positive value indicates a bias towards high-fat foods.

Analysis of the implicit wanting fat appeal bias (see Figure 3) revealed that the low satiety phenotype had a greater bias for high-fat foods when hungry across all conditions compared to the high satiety phenotype who exhibited a greater bias for low-fat foods (20% RMR; (*t* (16) = 4.82, *p* < 0.001, *d* = 2.4), 25% RMR; (*t* (16) = 2.25, *p* < 0.04, *d* = 1.1), 30% RMR; (*t* (16) = 2.73, *p* < 0.02, *d* = 1.4) and 35% RMR; (*t* (16) = 2.19, *p* < 0.05, *d* = 1.1).

### 3.7. Craving for Food (CoEQ)

The low satiety phenotype (M: 48.2, SD: 19.7) scored lower on the Craving Control subscale of the CoEQ (*t* (16) = 2.41, *p* < 0.03, *d* = 1.2) compared to the high satiety phenotype (M: 73.2, SD: 24.1). There were no differences between the low and high satiety phenotype on the Craving for Sweet (*t* (16) = 0.294, *p* > 0.05), Craving for Savoury (*t* (16) = 1.27, *p* > 0.05) or Positive Mood (*t* (16) = 1.49, *p* > 0.05) subscales of the CoEQ.

## 4. Discussion

The failure of some individuals to generate or detect adequate signals to stop eating has frequently been reported in clinical contexts. The current research aimed to determine the reliability of the low satiety phenotype and the consistency of the SQ under different energy loads, and secondly to examine a behavioural, psychological and physiological risk profile for overeating in normal and overweight women systematically identified as being low or high in satiety responsiveness. The current study found that a low SQ was associated with a higher resting metabolic rate, a greater implicit wanting for high fat foods and higher scores on the TFEQ Disinhibition subscale. Using the SQ as a categorical variable, the findings of the current study indicated that it is possible to identify women who reliably experience weak or strong satiating efficiency under controlled laboratory conditions. The current study did not find that low satiety responsiveness was associated with a higher BMI. It may be that weakened satiety responsiveness becomes more important for weight gain later in life, something that should be investigated in future research. It is important to note that out of the thirty participants who completed the study, twelve could not be reliably categorised suggesting that it may be beneficial for future research to identify the low satiety phenotype based on more than one assessment of satiety responsiveness. When these twelve individuals were included in the analysis they were not statistically distinguishable from the low or high satiety phenotypes but responded in a manner more similar to that of the high rather than the low satiety phenotype. Therefore, it appears that women who exhibit consistently low satiety responsiveness are of particular interest for further investigation as a group at increased risk of overeating and potential future weight gain.

Specifically, we found that the low satiety phenotype had greater TFEQ disinhibition scores compared to the high satiety phenotype. Disinhibition reflects the tendency to eat opportunistically [6] and greater levels of disinhibition have been consistently associated with increased *ad libitum* energy intake [22,23] and increased propensity for weight gain [24,25]. In addition and consistent with our findings, several studies have found that disinhibition is associated with weak satiety responsiveness [26,27]. For example, Finlayson *et al*. [26] demonstrated that higher levels of disinhibition were associated with lower satiating efficiency of sweet and savoury preloads.

Further to this, we demonstrated that the low satiety phenotype consumed significantly more energy in the *ad libitum* lunch test meal. This finding is consistent with previous research that has shown a low SQ in response to a standardised test meal is negatively associated with energy intake under laboratory and free-living conditions [8,9]. A possible explanation for this higher energy intake may be that the low satiety phenotype also had a higher resting metabolic rate (and a higher fat free mass, the largest contributor to resting metabolic rate) compared to the high satiety phenotype. The notion of energy expenditure as a metabolic driver of energy intake while not a new concept in the study of energy balance and appetite control [28] has recently been reconsidered with the suggestion that resting metabolic rate may be a functionally relevant biological signal for energy need and therefore act as a regulator of appetite control and food intake [29,30,31]. Therefore, the greater resting metabolic rate observed in the low satiety phenotype may reflect a greater biologically based drive to eat. However, it is important to note that the four fixed energy breakfasts were calibrated individually for each subject based on their own measured energy requirements. Therefore, the weak satiety response to the breakfast meals exhibited by the low satiety phenotype was not simply a function of their relative energy needs not being accounted for.

Despite this increase in energy intake we did not find that the low satiety phenotype reported greater levels of hunger, desire to eat, prospective consumption or lower levels of fullness across the test day. Interestingly, however we found that the low satiety phenotype consistently reported lower levels of fasting hunger, desire to eat and prospective consumption. All subjects in the current study were regular breakfast consumers and based on the findings from energy intake it may be anticipated that the low satiety phenotype would have higher levels of baseline hunger, desire to eat and prospective consumption. It may be that following a period of fasting the low satiety phenotype are relatively poor at detecting their true appetite sensations, which is consistent with the findings of Barkeling *et al.* (2007) [5]. However, it is important to note that habitual breakfast consumption was not assessed so it is not possible to discern whether the low satiety phenotype were habitually small breakfast consumers which may account for the lower level of fasting hunger.

To measure hedonic risk factors for overeating in the low satiety phenotype, we examined liking and wanting appeal bias for high fat *versus* low fat foods. We found that compared to the high satiety phenotype, the low satiety phenotype consistently exhibited a greater wanting appeal bias for high-fat foods (*i.e.*, they chose high-fat foods more frequently and faster than they chose low-fat foods) across all conditions. Previous research has shown that increased wanting for high-fat food is associated with greater compensatory eating behaviours following exercise, greater binge eating tendencies, and greater overall energy intake [32,33,34,35]. In contrast, the high satiety phenotype had a greater appeal bias for low-fat foods, a preference that may be protective against creating a positive energy balance. Indeed, research has demonstrated that a greater preference for low-fat foods is negatively associated with energy intake under laboratory and free-living conditions (assessed using 24-h dietary recall) [36]. In addition, the low satiety phenotype reported experiencing lower control over their cravings over the previous seven days. The tendency to experience greater food cravings has been associated with greater BMI [37,38]. These findings are the first to suggest that the low satiety phenotype may be characterised by hedonic risk factors for overconsumption.

Although the present study carried a number of strengths there are some limitations to consider. The repeated characterisation of the low and high satiety phenotypes, while a strength of the current study, resulted in the sample size to be small and therefore the novel findings of the current study stand to be replicated in a larger sample. However, it is important to note that the findings of the current study were very consistent with that of previous work and the effect sizes reported were medium to large which supports the strength of the findings. One methodological limitation of the present study was the energy density of the food items provided in the *ad libitum* lunch. The energy density of some of the food items provided was quite high (~1.4–2 kcal/g), which resulted in unusually high *ad libitum* lunch intakes.

The energy density of the meal provided is an important consideration for future research. As the current study only examined satiety responsiveness in female subjects the findings may not be generalisable to males. In addition, the cross sectional nature of the current study makes it difficult to infer the specific causes behind the low satiety phenotype. For instance, it is not known whether eating behaviour traits such as Disinhibition lead to weakened satiety, or whether weak satiety signaling leads to altered eating behaviour traits like more opportunistic eating such as snacking. While participants were asked to not engage in physical activity for 24-h prior to each experimental session, participants’ habitual physical activity levels were not assessed and so it is not known whether the low satiety phenotype were more or less physically active than the high satiety phenotype. If the low satiety phenotype expend more or less energy in structure and non-structured physical activity this may have an impact on the degree of satiety achieved from a given meal size. This limitation should be addressed in future work. The current study identified the low satiety phenotype based on the response to four fixed energy breakfasts that differed in their energy load, energy density (the higher RMR conditions were more energy dense) and macronutrient content (the 25%, 30% and 35% RMR breakfasts would have had a higher fat and protein content because of the greater almond content). This could be improved upon by holding the macronutrient content of the fixed energy breakfast constant. While it is beyond the scope of the current paper, future work could manipulate macronutrient composition to examine whether the low satiety phenotype exhibit a weakened satiety response to meals that have been designed to have a high satiating impact such as those high protein or low in fat (see [39]), which may present as an effective nutritional strategy for such individuals. Finally, while previous research has reported that the low satiety phenotype are characterised by an attenuated cortisol response to a test meal [10], future work should explore the possibility of further potential biomarkers that may underlie the low satiety phenotype’s weakened satiety response.

## 5. Conclusions

To conclude, the current study repeatedly identified individuals as high or low in satiety responsiveness and examined their behavioural, psychological and physiological risk profile for overeating and obesity. It was confirmed that the SQ could be used to identify a proportion of individuals who exhibited reliably weak satiating efficiency, and that these individuals display traits and preferences that would increase their susceptibility to overeating and future weight gain.
